# Endostatin induces normalization of blood vessels in colorectal cancer and promotes infiltration of CD8+ T cells to improve anti-PD-L1 immunotherapy

**DOI:** 10.3389/fimmu.2022.965492

**Published:** 2022-10-24

**Authors:** Xiao-Dong Chu, Hui Bao, Yu-Jian Lin, Ruo-Xi Chen, Yi-Ran Zhang, Ting Huang, Jia-Shuai He, Shu-Chen Huangfu, Yun-Long Pan, Hui Ding

**Affiliations:** ^1^Department of General Surgery, First Affiliated Hospital of Jinan University, Guangzhou, China; ^2^Department of Plastic Surgery, First Affiliated Hospital of Jinan University, Guangzhou, China; ^3^Department of Clinical Pathology, First Affiliated Hospital of Jinan University, Guangzhou, China

**Keywords:** colorectal cancer, recombinant human endostatin, PD-L1 inhibitor, tumor vascular normalization, CD8+ T cells

## Abstract

**Introduction:**

The purpose of this study was to evaluate recombinant human endostatin (rHE)-induced normalization of the tumor vasculature in colorectal cancer (CRC) and to evaluate the therapeutic effects of combined treatment with rHE and a programmed death ligand-1 (PD-L1) inhibitor.

**Methods:**

A mouse subcutaneous tumorigenesis model was established to evaluate the antitumor effects of endostatin combined with a PD-L1 inhibitor on CRC. Intravoxel incoherent motion diffusion-weighted magnetic resonance imaging (IVIM-DW MRI) was used to evaluate changes in the intratumor microcirculation in response to combined treatment with endostatin and a PD-L1 inhibitor. The infiltration density and function of CD8+ T cells in tumors were evaluated using flow cytometry. Finally, clinical specimens were used to evaluate the expression area of tumor vascular pericytes and CD8+ T cells in tumor tissues.

**Results:**

The antitumor effects of endostatin combined with a PD-L1 inhibitor were significantly greater than those of endostatin or a PD-L1 inhibitor alone. On the ninth day of intervention, the endostatin group showed significantly higher pseudo diffusion parameter (D*) and microvascular volume fraction (F) values in tumors than those in the control group or PD-L1 group. After 27 days of intervention, the endostatin groups showed significantly lower levels of vascular endothelial growth factor (VEGF) and transforming growth factor (TGF)-β than those in the control group. Treatment of CD8+ T cells with endostatin for 24 h did not alter the expression levels of markers of reduced T-cell activity. However, endostatin reversed the VEGF-mediated inhibition of the secretion of interferon (IFN)-γ from T cells. The results in CRC clinical samples showed that treatment with endostatin induced significantly higher infiltration of CD8+ T cells compared with treatment that did not include endostatin. Furthermore, the expression area of pericytes was significantly positively related to the infiltration density of CD8+ T cells and overall survival time.

**Conclusion:**

Endostatin improved the antitumor effects of PD-L1 inhibitors on CRC, significantly increased the activity of CD8+ T cells, and synergistically improved the tumor treatment effect of the two inhibitors.

## Introduction

Colorectal cancer (CRC) is the third most common cancer worldwide and is one of the leading causes of cancer-related death (1). The American Cancer Society (ACS) released a statistical report on colorectal malignant tumors in the United States in 2020 (2). CRC is the third most common cause of cancer death in men and women in the United States. Increasingly sophisticated surgical procedures, targeted therapies, innovative antitumor vascular therapies, and emerging immunotherapies have enhanced the survival rates of some patients with advanced malignant colorectal tumors (3–5). However, the prognoses of some patients with targeted drug resistance and high tumor invasiveness are poor (6).

Abnormalities in the tumor vasculature lead to hypoxia, acidosis, and high interstitial pressure in the tumor microenvironment (TME) (7, 8). Hypoxia and acidosis promote immunosuppression through accumulation, activation, and extension of T cells (9–11). This process results in the recruitment of inflammatory monocytes and tumor-associated macrophages (TAMs), leading to transformation of TAMs from the M1 phenotype to the M2 phenotype (12–14). Inhibition of dendritic cell (DC) maturation leads to reduced antigen presentation and activation of tumor-specific cytotoxic T lymphocytes (CTLs) (15, 16). The proliferation of abnormal endothelial cells (ECs) occurs in response to the immunosuppressed microenvironment (17, 18). In addition, activation of the programmed death-1 (PD-1)/programmed death ligand-1 (PD-L1) pathway and upregulation of PD-L1 in TAMS occur in the TME, resulting in immune escape (19, 20). Tumor-infiltrating CTLs upregulate PD-1, marking it as dysfunctional or “depleting” and restricting its cytotoxic potential to tumor cells (21, 22). In addition, vascular endothelial growth factor (VEGF)-A, a proangiogenic molecule produced by tumor cells, plays a key role in the development of an immunosuppressive microenvironment. Blockade of VEGF-A has been shown to enhance the activation of CD8+ T cells within the tumor, resulting in increased ability to produce cytokines (23). These processes result in abnormal tumor angiogenesis, and vascular abnormalities are an immunosuppressive TME. Preclinical studies have shown that combination treatment with vascular normalization drugs with inferences alleviating T-cell functional blockade (24–26). For example, immune checkpoint blockers (ICBs) with anti-PD-1 antibody improve the degree of tumor control achieved with anti-ANG2-VEGF antibody A2V in various cancer models (21, 27). Preclinical and clinical evidence has suggested that anti-VEGF therapy builds a time window for vascular normalization, during which the delivery of oxygen, radiosensitizers, immunostimulators, and other therapeutic agents is improved (28).

Pericytes are mesenchymal cells that stabilize and wrap capillaries. They are embedded in the basement membrane of small blood vessels and affect ECs by secreting endothelial growth factors and matrix metallopeptidase (MMP) inhibitors. Pericytes also stabilize EC junctions to limit vascular permeability. The lack of stable pericyte–endothelial interactions in tumors inhibits angiogenic sprouting, resulting in dysfunctional vascular networks characterized by endothelial proliferation, defective cell junctions, and vascular leakage. ECs secrete platelet-derived growth factor subunit B (PDGFB) to promote pericyte recruitment to the tumor vasculature. Kinase inhibitors that block both vascular endothelial growth factor receptor (VEGFR) and platelet-derived growth factor receptor (PDGFR), such as sunitinib and sorafenib, suppress tumors better than VEGFR inhibitors alone. These findings suggested that pericytes may provide critical prosurvival cues for angiogenesis (29).

Endostatin is a multitarget antiangiogenic drug that exerts therapeutic effects through the regulation of EC surface protein expression and cell signaling pathways at the molecular level and regulation of the TME (30). Endostatin can directly bind to VEGFR2 and inhibit its phosphorylation to block the VEGF-VEGFR2 pathway, resulting in tumor vascular normalization (31). However, the efficacy of antiangiogenic therapy using recombinant human endostatin (rHE) combined with immunotherapy has yet to be evaluated. Clinical data and elucidation of mechanisms of action are needed.

## Materials and methods

### Cell strain and cell culture

CT26 murine CRC cell lines were provided by the Cell Bank of the Chinese Academy of Sciences and were cultured in 90% Roswell Park Memorial Institute (RPMI) 1640 medium + 10% fetal bovine serum (FBS) + 1% double antibodies (streptomycin and penicillin) + 1% non-essential amino acid + 1% L-glutamine at 37°C in a 5% CO_2_ cell incubator.

### Mouse subcutaneous xenograft model and group intervention

Twenty-five BALB/C mice aged 5–7 weeks weighing 18–26.5 g were provided by the Guangdong Medical Laboratory Animal Center. The mice were subcutaneously inoculated with CT26 cells. The mice were housed under standard conditions, and they were observed daily. Tumors were allowed to grow. Two weeks after inoculation with tumor cells, tumor growth was observed in all 25 inoculated mice, which indicated a tumorigenesis rate of 100%. The long diameter (A) and short diameter (B) of the tumor were measured on the body surface using a Vernier caliper to calculate the tumor volume using the following equation: V = 0.532ab2. Tumors that were too large or too small were excluded. Twenty mice were included in the study. The 20 mice were divided into four groups using the random number method. There were no group differences in average tumor volume among the groups. The treatment groups were as follows: 1) Endostar group: On days 3, 6, 9, 12, 15, and 18 after group assignment, mice were administered rHE (Endostar, Simcere) (5 mg/kg) subcutaneously; 2) PD-L1 inhibitor group: Subcutaneous injection of a PD-L1 inhibitor (anti-mouse PD-L1 monoclonal antibody Clone 10F.9G2, BioXcell) was administered at a dose of 10 mg/kg every 3 days for a total of six doses; 3) Endostar+PD-L1 inhibitor group (Endostar+PD-L1): The mice received subcutaneous injections of Endostar (5 mg/kg) combined with a PD-L1 inhibitor (10 mg/kg) once every 3 days for a total of six doses; and 4) Control group (Ctrl): Mice were injected subcutaneously with an equal volume of normal saline at the same times as the mice in the treatment groups. Intravoxel incoherent motion diffusion-weighted magnetic resonance imaging (IVIM-DWI MRI) was performed on Days 0, 9, and 18 after intervention. On Day 27, all mice were sacrificed by neck dissection. All animal experiments were performed in accordance with the Guidelines of the Ethics Committee for Animal Experiment of Jinan University. The Ethics Committee for Animal Experiment of Jinan University approved the study proposal.

### Intravoxel incoherent motion diffusion-weighted magnetic resonance imaging

Prior to MRI, the mice were anesthetized (intraperitoneally) with 2% sodium pentobarbital and remained sedated for the duration of the magnetic resonance scan. Experimental magnetic resonance scans were performed using an Agger Medical System equipped with an eight-channel body coil and a 1.5T Signa HDXTL.5T superconducting MRI system. T2-weighted images were obtained using fast spin echo (FSE) sequences with the following imaging parameters: 0.2-mm layer gap, 2.0-mm layer thickness, Time Echo (TE) 91.8 ms, Time Repeat (TR) 4,000 ms, field of view (FOV) 10 cm × 7 cm, and matrix size 128 × 96. The following coefficients were obtained using IVIM: fast diffusion coefficient (D*) fast Apparent diffusion coefficients (ADC), slow diffusion coefficient (D) slow ADC, and perfusion fraction (f) fast adc. D* is the pseudo diffusion coefficient obtained from the microcirculation, which reflects perfusion. D is the true diffusion parameter of water molecules. F is the microvascular volume fraction, which is the percentage of perfusion, and reflects blood flow. The image with the largest tumor cross-section was selected on T2W, and the tumor boundary was manually drawn to delineate the region of interest.

### Immunofluorescence detection of microvessel density and tissue hypoxia expression

Paraffin-embedded tumor tissues were sectioned to a thickness of 5 μm and dewaxed to water. The primary antibodies used included anti-mouse CD31 (AF 3628, R&D Systems) and rabbit anti-mouse Hypoxia-inducible factor (HIF)-1α (AB179483, Abcam) and the corresponding fluorescent secondary antibodies. Antibodies were added dropwise into the water blockade ring to cover the tissue. Slice images were collected using a confocal fluorescence microscope (Confocal/Living Cell Workstation).

### Flow cytometry detection

The density of infiltrating CD8+ T cells in the tumors and the expression levels of interferon (IFN)-γ were evaluated using flow cytometry. After the mice were sacrificed, fresh subcutaneous xenograft tissue samples were taken to obtain tumor-infiltrating lymphocytes. Tumor-infiltrating lymphocytes and peripheral blood lymphocytes were suspended, and 1 μl of Fluorescein isothiocyanate (FITC) anti-mouse CD8 (alpha subunit, CD8a, Ly-2, 11-0081-81, eBioScience) and phycoerythrin (PE) anti-mouse IFN-γ (Clone Number: XMG 1.2, E-AB-F11101UD, Elabscience) were added into the tube. After routine operation, the supernatants were collected and flow cytometry was performed.

### Isolation of mouse T cells

The mice were sacrificed by cervical dislocation. The abdomens of the mice were aseptically dissected, and the spleens were removed, sieved through a cell sieve, and crushed. After the supernatant was removed, red blood cell lysates were added and the supernatant was removed following centrifugation. The cells were cultured and resuspended to remove insoluble tissue fibers, and the cells in the suspension were counted. Then, CD8+ T cells were isolated using the EasySep™ mouse CD8+ T-cell enrichment kit (Stem Cell Technologies, 19853).

### ELISA testing

ELISA kits for mouse VEGF, interleukin (IL)-6, IL-10, and transforming growth factor (TGF)-β were purchased from Zhenke Biology, Shanghai. The levels of VEGF, IL-6, IL-10, and TGF-β were detected in serum. A mouse IFN-γ ELISA kit, purchased from Soleil, was used to determine levels of secreted IFN-γ in cell supernatants.

### Cell treatments

Mouse CD8+ T cells were plated and treated with recombinant mouse VEGF (Pepro Tech) with or without rHE. Cell supernatants were collected and centrifuged, and the supernatants were used for analysis. The expression levels of CD8+ T-cell markers PD-1, cytotoxic T lymphocyte-associated antigen 4 (CTLA-4), T-cell immunoglobulin and mucin domain 3 (TIM3), Lymphocyte activation gene-3 (LAG3), and T cell immunoglobulin and ITIM domain (TIGIT) were detected using RT-qPCR. We used Glyceraldehyde-3-phosphate dehydrogenase (GAPDH) as the internal reference, and the primers were all designed by Primer Premier 6.0 primer design software and synthesized by Biological Engineering (Shanghai) Co., Ltd. T-cell proliferative capacity was measured using the 3-(4,5-dimethylthiazol-2-yl)-2,5-diphenyltetrazolium bromide (MTT) assay. After CD8+ T cells were plated on 96-well plates, they were placed in the incubator in MTT buffer for 4 h. Then, MTT lysate was added to each well, and the plates were shaken gently and incubated overnight. The plates were read at 570 nm. The mean and standard deviation were calculated and plotted.

### Clinical cases and specimen processing

A total of 37 paraffin sections from patients with CRC who were treated surgically in the First Affiliated Hospital of Jinan University from 1 January 2013 to 1 January 2016 were retrospectively analyzed. All patient specimens were confirmed as CRC by the Department of Pathology, and each patient had complete clinical data. The study included 22 men and 15 women aged 47–66 years, and the average age was 56.4 ± 4.5 years. According to the TNM staging criteria of the American Joint Committee on Cancer (AJCC) eighth edition of CRC, there were 14 cases in stage II, 19 cases in stage III, and four cases in stage IV. The patients were divided into an endostatin intervention (rHE) group and a non-endostatin intervention (non-rHE) group. Seventeen patients were treated with neoadjuvant chemotherapy combined with endostar intervention prior to surgery. All surgically resected colon cancer tissues were embedded in paraffin using an automatic biological tissue embedding machine. Paraffin-embedded CRC tissue and paracancerous normal colon tissue were selected using patient clinical data. Serial tissue sections (thickness of 5 μm) were prepared, and at least five sections were cut consecutively for each tissue. All patients signed an informed consent for pathological examination. This study met the requirements of the Hospital Ethics Committee and was approved after review.

Immunohistochemistry was used to determine the expression levels of Alpha-smooth muscle actin (α-SMA), CD8+ T cells in the peripheral cells of clinical tissues, and PD-L1 in mouse tissues. After sections are baked and deparaffinized, antigen retrieval is performed and endogenous peroxidase is blocked. After blocking, sections were incubated with primary antibodies, then incubated with secondary antibodies. Then, Diaminobenzidine (DAB) color developing solution was added dropwise. Positive cells were brownish yellow, the nuclei were counterstained, the slices were dehydrated, and images were collected for analysis. Photographs were imported into Image Pro Plus V6.0 software for further analysis.

### Statistical analysis

Statistical analyses were performed using GraphPad Prism (Version 5.01). Normal distributed data were expressed as $\bar{x}$ s, and analyses with two groups (kindness intervention group and non-kindness intervention group) were subjected to t-tests. Data with non-normal distributions were expressed as medians (interquartile range), and non-parametric tests were used to compare two groups. Categorical data were shown as percentages, and comparisons were performed using the chi-square test or Fisher’s exact test. Spearman correlation was used to analyze the marker coverage rate of pericolonic cancer cells and the infiltration density of CD8+ T cells in CRC tumor tissues. The association of CD8+ T-cell density on the prognosis of patients with CRC was evaluated using Kaplan–Meier survival analysis. The test criterion was α = 0.05. When the result is P< 0.05, it is considered that there is a significant difference. All data were processed by SPSS 20.0 statistical software.

## Results

### Evaluation of the antitumor effects of endostatin combined with a programmed death ligand-1 inhibitor and monitoring of intratumor microcirculation

**Figure 1:**
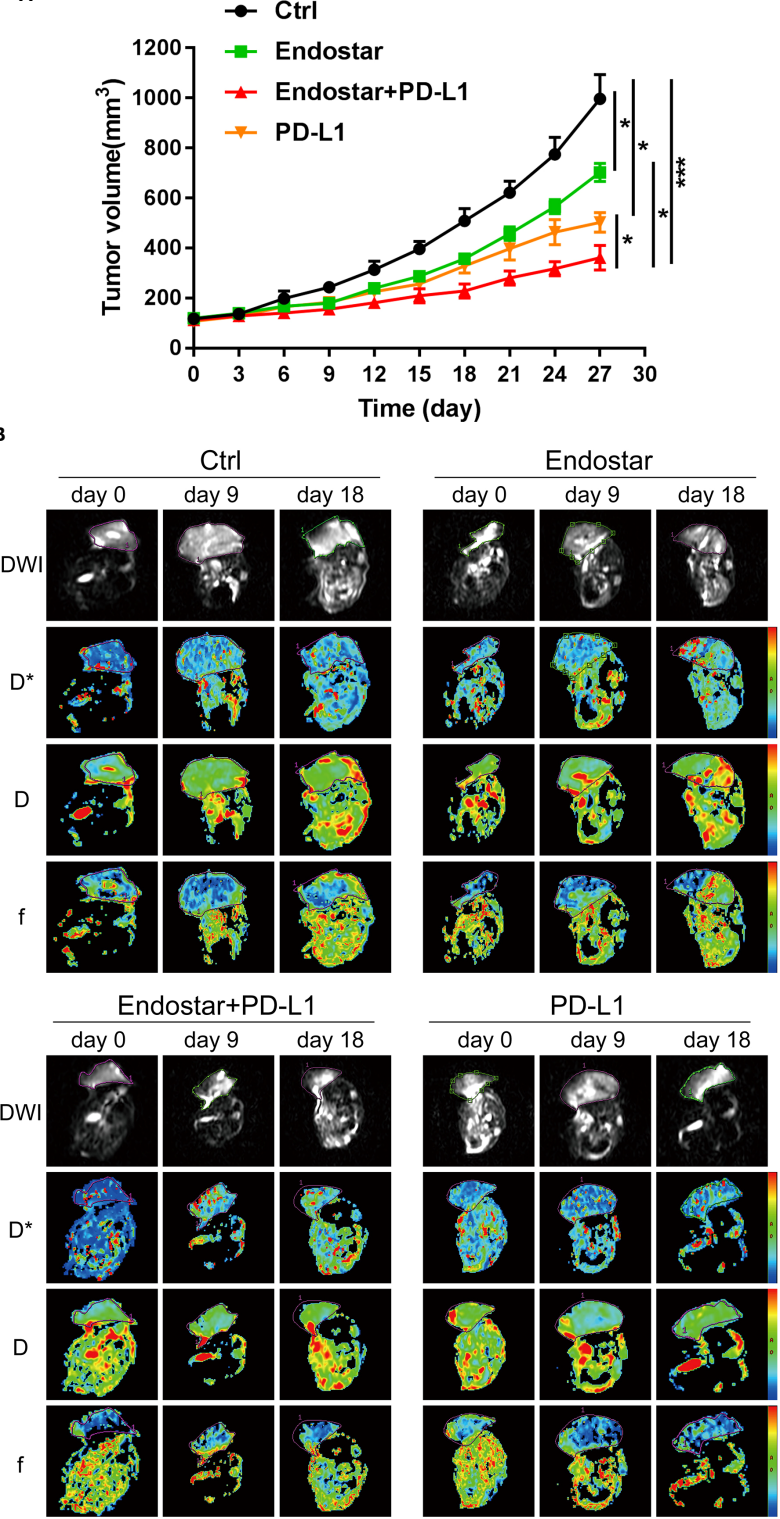
Evaluation of the antitumor effect of Endostar combined with programmed death ligand-1 (PD-L1) inhibitor. **(A)** The effect of Endostar combined with PD-L1 inhibitor on the xenograft volume of CT26 mice (*P< 0.05; ***P< 0.001). **(B)** Intravoxel incoherent motion diffusion-weighted magnetic resonance imaging (IVIM-DWI MRI) is used to evaluate the tumor microcirculation of colorectal cancer (CRC) intervention mice in Endostar, Endostar+PD-L1, and PD-L1 groups. IVIM-DWI MRI was performed before the intervention and on the ninth and 18th days after the intervention.

The mouse xenograft model was constructed using CT26 cells. The antitumor effects of Endostar, a PD-L1 inhibitor, and Endostar combined with a PD-L1 inhibitor provided significantly better therapeutic effects than the control treatment (P< 0.05). The antitumor effects in the Endostar+PD-L1 inhibitor group were significantly better than those observed in response to Endostar or a PD-L1 inhibitor alone (P< 0.05), as shown in **Figure 1A**. These outcomes showed that Endostar+PD-L1 inhibitor was superior to PD-L1 alone.

**Figure 2:**
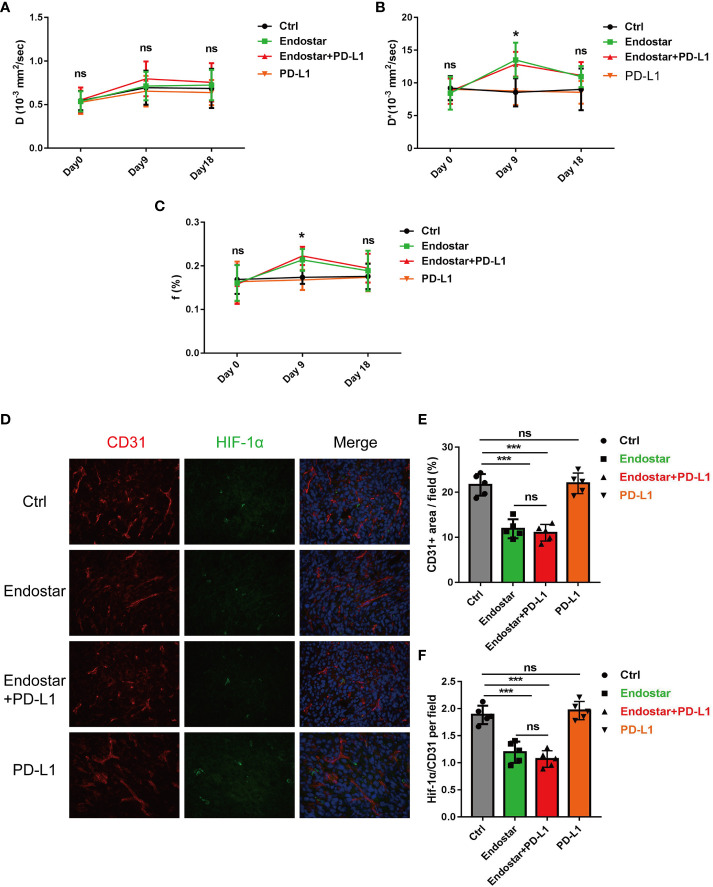
Changes of intravoxel incoherent motion diffusion-weighted magnetic resonance imaging (IVIM-DWI MRI)-related indexes in Endostar, Endostar+programmed death ligand-1 (PD-L1), and PD-L1 groups after intervention of colorectal cancer (CRC) mouse tumor. **(A)** Comparison of real diffusion coefficients **(D)** of water molecules in the four groups of mouse tumors at different times. **(B)** Comparison of pseudo diffusion coefficients (D*) of four groups of mouse tumors at different times. **(C)** The microvascular volume fraction **(F)** of water molecules in the tumor of mice in group was compared at different times. **(D–F)** Comparison of microvessel density and tissue hypoxia parameters of CRC by different interventions (*P< 0.05, ***P< 0.001). ns, not significant, P>0.05.

Following drug intervention, IVIM-DWI MRI scans were performed prior to intervention (Day 0) and on Days 9 and 18 after treatment. **Figure 1B** shows the images of the mice in each group. No significant differences were observed in the true diffusion parameter (D) of water molecules, the pseudo diffusion parameter (D*), or the microvessel volume fraction (F) of water molecules in the tumors of the four groups prior to the intervention. On the ninth day of intervention, there was no significant difference in the true diffusion coefficient (D) of water molecules of tumors between the four groups (P > 0.05). The pseudo diffusion coefficient (D*) and microvessel volume fraction (F) of the tumors in the Endostar group and the Endostar+PD-L1 group were significantly higher than those in the control group and the PD-L1 group (P< 0.05). On Day 18 after the intervention, there were no significant differences in the true diffusion parameter (D) of water molecules, the pseudo diffusion parameter (D*) of the tumors, or the microvessel volume fraction (F) of water molecules among the four groups of mice (**Figures 2A–C**).

### Effects of endostatin combined with a programmed death ligand-1 inhibitor on hypoxia in colorectal cancer blood vessels and tissues

After the mice were sacrificed, immunofluorescence analysis of the tumor tissues (**Figure 2D**) showed that Endostar and Endostar+PD-L1 treatment resulted in significantly lower microvascular densities than those in the control group or the PD-L1 group (P< 0.05). No differences were observed between the Endostar and Endostar+PD-L1 groups (**Figure 2E**). The tumor hypoxia areas (HIF-1α) in the Endostar and Endostar+PD-L1 groups were significantly lower than those in the control group or the PD-L1 group (P< 0.05). There were no differences between the Endostar and the Endostar+PD-L1 groups (P > 0.05) (**Figure 2F**).

### Effect of Endostar combined with a programmed death ligand-1 inhibitor on density and function of infiltrating CD8+ T cells

**Figure 3:**
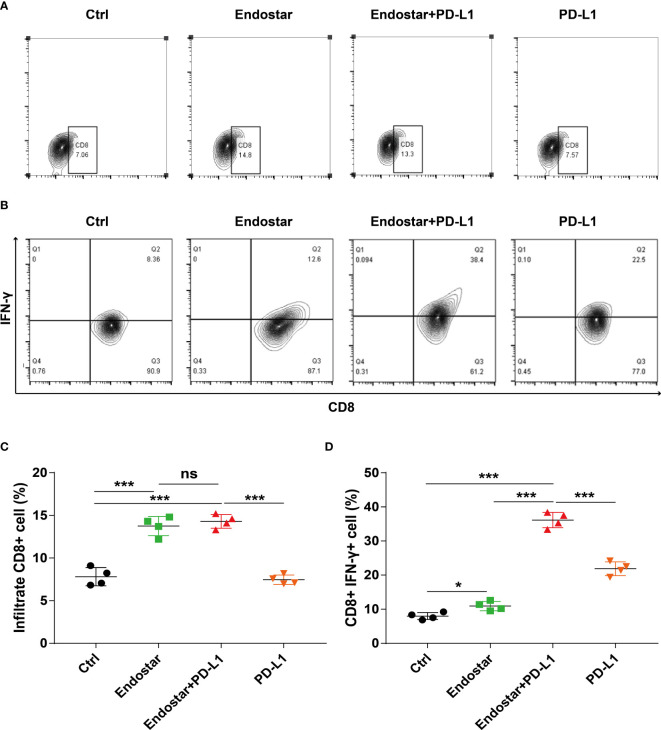
Density and function of CD8+ T cells infiltrating colorectal cancer detected by flow cytometry when recombinant human endostatin is combined with a programmed death ligand-1 (PD-L1) inhibitor. **(A)** Flow cytometry is used to detect the infiltration density of CD8+ T cells in the tumors of the four groups of mice. **(B)** The proportion of interferon (IFN)-γ-positive cells of CD8+ T cells in the tumors of the four groups of mice is detected by flow cytometry. **(C)** Comparison of the infiltration density of CD8+ T cells in the tumors of the four groups of mice. **(D)** Comparison of the proportion of IFN-γ-positive CD8+ T cells in the tumors of the four groups of mice (*P<0.05; ***P< 0.001). ns, not significant, P>0.05.

Mouse tumor tissues were collected and processed to produce suspensions of cells. Flow cytometry was used to evaluate the infiltration density of CD8+ T cells in the tumors (**Figures 3A, B****)**. The Endostar and Endostar+PD-L1 groups showed significantly higher infiltration densities of CD8+ T cells than those of the control or PD-L1 group (P< 0.05). The infiltration densities of CD8+ T cells in the Endostar and Endostar+PD-L1 groups did not differ (**Figure 3C**, P > 0.05). Analysis of IFN-γ secretion from infiltrated CD8+ T cells showed that the CD8+ T cells in the Endostar, Endostar+PD-L1, and PD-L1 groups secreted more IFN-γ than those in the control group (P< 0.05). Moreover, CD8+ T cells in the Endostar+PD-L1 group secreted significantly more IFN-γ than those in the Endostar or PD-L1 group (**Figure 3D**, P< 0.05).

### Comparison of the expression levels of serum vascular endothelial growth factor (VEGF), interleukin (IL)-6, IL-10, and transforming growth factor (TGF-β) and the expression of programmed death ligand-1 in tumor tissues of mice before and after the intervention

**Figure 4:**
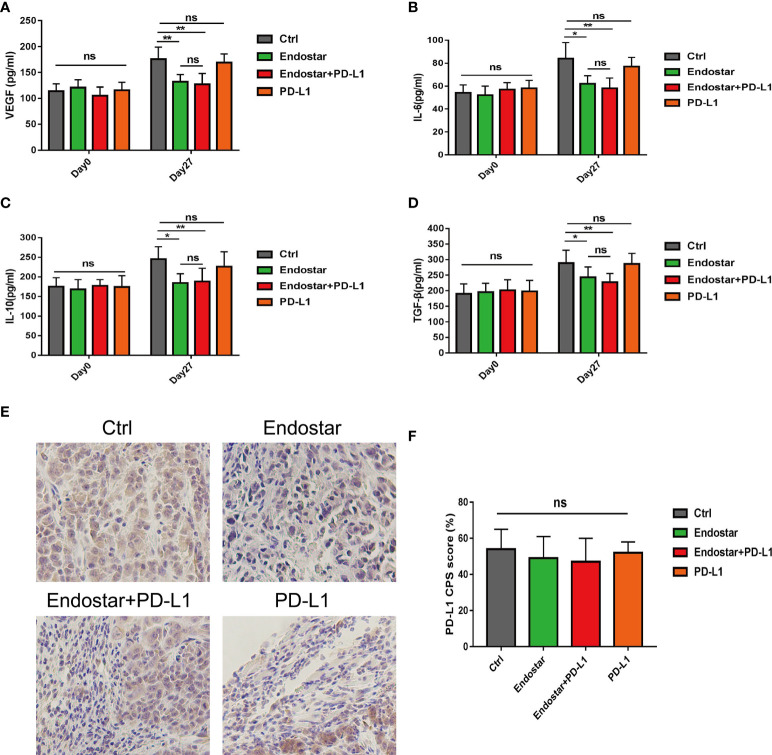
Comparison of serum vascular endothelial growth factor (VEGF), interleukin (IL)-6, IL-10, and transforming growth factor (TGF)-β levels between before and 27 days after intervention. **(A)** Serum vascular endothelial growth factor (VEGF), levels in the control, Endostar, Endostar+programmed death ligand-1 (PD-L1), and PD-L1 groups. **(B)** Comparison of serum interleukin (IL)-6 levels among the four groups. **(C)** Comparison of serum interleukin (IL)-10 levels among the four groups. **(D)** Comparison of serum transforming growth factor (TGF-β) levels among the four groups. After 27 days of intervention, the expression levels of PD-L1 in tumors of mice in each group were compared. **(E)** programmed death ligand-1 (PD-L1) expression levels of tumors in mice of the immunohistochemical control (Ctrl), Endostar, Endostar+PD-L1, and PD-L1 groups (×400). **(F)** Comparison of combined positive score (CPS) scores of PD-L1 expression among the different groups of mouse tumors (ns, Not significant; P > 0.05). (*P<0.05, **P<0.01).

Serum levels of VEGF, IL-6, IL-10, and TGF-β did not differ among the groups before the intervention (P > 0.05). After 27 days of intervention, the Endostar and Endostar+PD-L1 groups had significantly lower levels of VEGF, IL-6, IL-10, and TGF-β than those in the control or PD-L1 group (P< 0.05). However, no differences in VEGF, IL-6, IL-10, or TGF-β were observed between the Endostar and the Endostar+PD-L1 groups (**Figures 4A–D**, P > 0.05).

**Figure 4E** shows a representative diagram of PD-L1 expression of tumors in mice among the different groups. No differences were observed in PD-L1 expression levels (combined positive score (CPS) scores) in tumors of mice among the four groups (**Figure 4F**, P > 0.05).

### The effect of endostatin on VEGF-mediated T-cell depletion

**Figure 5:**
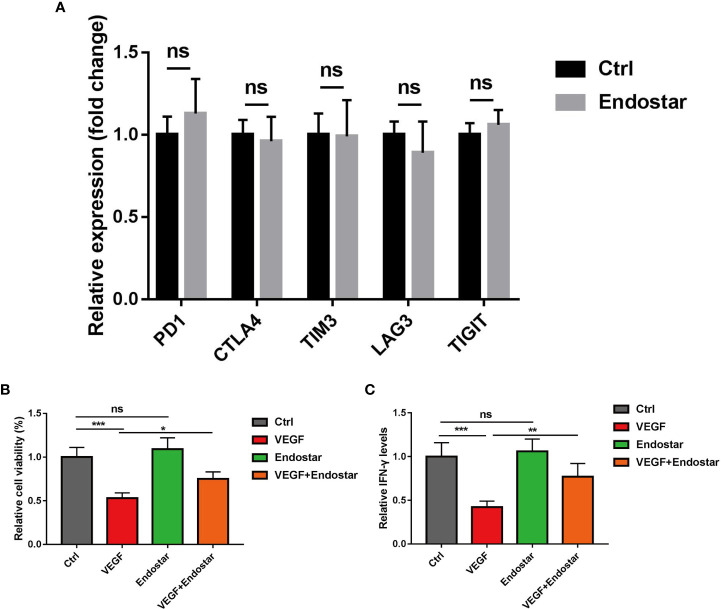
Effect of endostatin on vascular endothelial growth factor (VEGF)-mediated T-cell depletion. **(A)** Endostatin was used to interfere with CD8+ T cells in mice, and RT-qPCR was used to detect the expression changes of markers (PD-1, CTLA4, TIM3, LAG3, TIGIT) related to T-cell depletion in mice. **(B)** MTT assay Endostar was used to block the inhibition of VEGF on T cell-mediated cell proliferative activity under the intervention of VEGF. **(C)** ELISA test showed that Endostar was used to block the secretion of T cell-mediated IFN-γ by VEGF (ns, P > 0.05; *P< 0.05; **P< 0.01; ***P< 0.001).

After treatment of CD8+ T cells with Endostar for 24 h, RT-qPCR showed that the expression levels of PD-1, CTLA4, TIM3, LAG3, and TIGIT did not change in response to treatment (**Figure 5A**, P > 0.05).

In this study, treatment of mouse T cells with VEGF resulted in significantly reduced T-cell activity, as determined using the MTT assay (P< 0.05). No difference in T-cell activity was observed between mice in the Endostar group and the control group (P > 0.05). Treatment with VEGF and Endostar alleviated VEGF-mediated inhibition of T-cell activity (**Figure 5B**, P< 0.05). In addition, ELISA was used to determine the levels of IFN-γ secreted from T cells following each intervention. The results showed that Endostar reversed the VEGF-mediated inhibition of the secretion of IFN-γ from T cells (**Figure 5C**, P< 0.05).

### The correlation between the expression of pericytes and the infiltration density of CD8+ T cells in colorectal cancer

**Figure 6:**
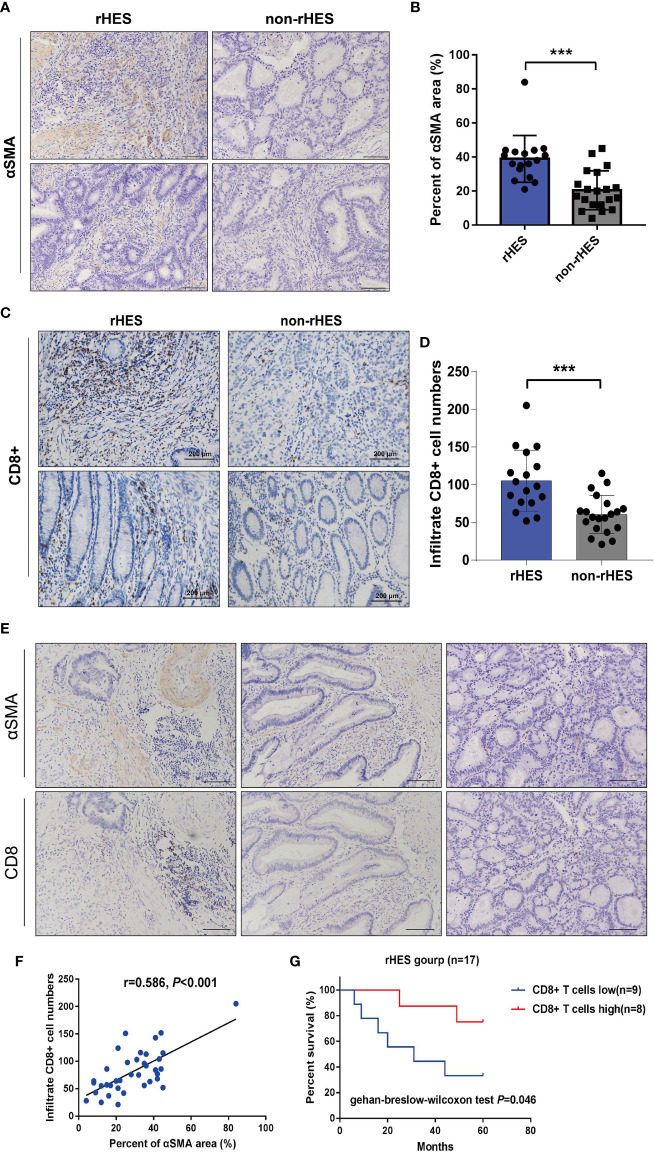
Relationship between the expression area of pericytes in the tumor tissue and infiltration density of CD8+ T cells in patients with colorectal cancer (CRC). **(A, B)** Comparison of the peritumoral cell expression areas in CRC patients treated with Endostar intervention. **(C, D)** Endorsement Intervention in colorectal cancer patients. **(E)** The correlation between the expression area of pericytes and the infiltration density of CD8+ T cells was examined by immunohistochemistry (×200). **(F)** Analysis of the correlation between the expression area of pericytes and the infiltration density of CD8+ T cells. **(G)** The effect of different CD8+ T-cell infiltration densities on the overall survival time of patients receiving Endostar intervention, ***P<0.001.

When the expression areas of pericytes in CRC tissues were compared between patients who received rHE before surgery and those who did not receive rHE, it was found that the expression levels of α-SMA markers in pericytes in the rHE group were significantly higher than those in the non-rHE group (**Figures 6A, B****)**, which indicated that rHE could be used to normalize the tumor vasculature.

The infiltration density of CD8+ T cells in CRC tumor tissues was compared between the rHE group and the non-rHE group. The rHE group showed significantly greater infiltration of CD8+ T cells labeled with α-SMA than the non-rHE group (**Figures 6C, D****)**. These results showed that Endostar could promote the infiltration of CD8+ T cells.

Serial sections were used to analyze the association between the expression area of pericytes and CD8+ T-cell infiltration density in CRC tissues. The expression of pericytes and CD8+ T-cell infiltration density in CRC tissues in the same tissue FOV are shown in **Figure 6E**. The expression area of pericytes in CRC tissues was significantly positively associated with CD8+ T-cell infiltration density (**Figure 6F**). The overall survival time of patients with a high CD8+ T-cell infiltration density was significantly longer than that in patients with a low infiltration density in the Endostar intervention group (**Figure 6G**).

## Discussion

The TME is typically characterized by hypoxia, low pH, and high interstitial hydraulic pressure, which can reduce the effectiveness of almost all types of anticancer treatments, including chemotherapy, radiotherapy, and immunotherapy. Therefore, normalization of tumor blood vessels can alter the TME, resulting in improved efficacy of immunotherapy (32, 33). Normalization of tumor blood vessels can reduce tumor vascular leakage, decrease the distortion and dilation of the vascular wall, normalize the basement membrane, and allow for homogeneous coverage of pericytes. Furthermore, alleviation of hypoxia in the TME can allow for improved transport of drugs into tumor tissues (34, 35). Endostar exerts an antiangiogenic effect by acting specifically on vascular ECs, resulting in the inhibition of proliferation and migration of vascular ECs, which leads to apoptosis (36, 37).

CD8+ T lymphocytes (CTLs) are the immune cells of choice for targeting tumors. CD8+ T lymphocytes become dysfunctional due to immune-related tolerance and suppression within the TME. Cancer-associated fibroblasts (CAFs) and regulatory T cells (Tregs) can form an immune barrier against CD8+ T cell-mediated antitumor immune responses. CD8+ T cells are thus primed and activated against effector CTLs during the tumor immune cycle to generate durable and potent antitumor immune responses. CD8+ T-cell priming acts as a facilitator between innate immune cells, including DCs and natural killer (NK) cells, and CD4+ T cells in adaptive immunity. When activated, effector CTLs infiltrate the core or invasive site of the tumor and play a key role in killing cancer cells (38).

PD-L1 is a key molecule that mediates reduced activity of T-cell killer tumor cells and is a target of PD-L1 inhibitors (39, 40). However, whether Endostar can regulate the expression level of PD-L1 in CRC has not been evaluated. A previous study (41) showed that T-cell failure can be induced by VEGF-A. VEGF-A induces the expression of the transcription factor thymocyte selection-associated high mobility group box (TOX) in T cells to boost the exhaustive given transcription program in T cells. Combined blockade of PD-1 and VEGF-A can restore the antitumor function of T cells and thus better control the microsatellite stable CRC.

In this study, we first evaluated the microcirculation in tumor tissues using voxel-based IVIM-DWI MRI. The TME is composed of blood vessels and lymphatic vessels, stromal cells, and resident and infiltrated immune cells. Our previous study using IVIM-DWI MRI showed that the D* value and F value could be used in place of Microvessel density (MVD), peripheral cell coverage, and intratumor perfusion rate to detect the vascular normalization and timing of vascular normalization. Our results showed that the perfusion rates in the tumor tissues of the rHE treatment group peaked on the ninth day of intervention, then gradually decreased. Furthermore, our experimental results showed that endostatin improved hypoxia in CRC tissues. Treatment with Endostar or Endostar+PD-L1 resulted in significant amelioration of hypoxia compared to that of the control group and the PD-L1 group (P< 0.05). No differences in hypoxia were observed between the Endostar and the Endostar+PD-L1 groups (P > 0.05).

Using animal models, we showed that coadministration of a PD-L1 inhibitor and Endostar improved anti-CRC efficacy and found that Endostar alone significantly increased IFN-γ secretion from tumor-infiltrating CD8+ T cells. *In vivo*, Endorsement can be achieved through improvement of the TME, including through reduced secretion of VEGF, IL-6, IL-10, and TGF-β. *In vitro*, we showed that VEGF inhibited the proliferation of mouse T cells and secretion of IFN-γ. In contrast, rHE reversed the VEGF-mediated T-cell inhibition. These results indicated that endostatin could inhibit tumor angiogenesis and reverse the immunosuppressive TME.

Pericytes play a role in tumor angiogenesis. They cover the surface of the basement membrane of vascular ECs and support and regulate vasoconstriction and relaxation under normal physiological conditions (42, 43). In multiple solid malignant tumors, the number of infiltrated CD8+ T cells was shown to be related to patient prognosis and response rate to immunotherapy (44–47). In our analysis of clinical samples, we found that pericyte coverage of tumor tissues in patients with CRC who received Endostar was significantly increased (as evidenced by labeled α-SMA). Furthermore, α-SMA could be used as a marker of pericyte labeling on the surface of basement membrane of new blood vessels. There was a significant positive association between the area of pericyte coverage and the infiltration density of CD8+ T cells in CRC tissues. The infiltration density of CD8+ T cells in patients who received Endostar was strongly associated with overall survival time. Increased infiltration of CD8+ T lymphocytes was associated with increased efficacy of PD-1/PD-L1 inhibitors. These results showed that Endostar induced increased CD8+ T-cell infiltration into CRC tumor tissues. In addition, Endostar induced increased secretion of IFN-γ, which indicated that Endostar improved the CD8+ T-cell tumor killing activity. These findings suggested that coadministration of anti-VEGF with PD-L1 exerted better antitumor effects than monotherapy. This dependence on VEGF-A production in the TME enhances the expression of inhibitory checkpoints involved in CD8+ T-cell exhaustion, rather than directly on CD8+ T cells (48). This phenomenon can be reversed through targeting of the VEGF-A–VEGFR axis, which provides a rationale for combination treatment with antiangiogenic and immunotherapeutic drugs to treat cancer.

The results of our preclinical and retrospective clinical research indicated that targeting the VEGF pathway has potential for the treatment of CRC. Administration of low-dose targeted VEGF pathway blockers prolonged the window of vascular normalization and transformed the immunosuppressive microenvironment into an immunosupportive environment. However, this normalization is transient, and the TME becomes hypoxic through upregulation of PD-L1 in tumor cells and TME stromal cells, including ECs, pericytes, and immune cells. Our results indicated that increased expression of pericytes on the surface of tumor blood vessels in CRC tissues could be indicative of a more complete tumor vascular structure. A more complete vascular structure may allow for better infiltration of immune cells, especially CD8+ T cells, which has potential clinical significance for improving tumor immunotherapy.

## Conclusion

Endostatin can induce vascular normalization of CRC. The level of vascular normalization of CRC tumors is closely related to the infiltration density of CD8+ T cells. The infiltration density of CD8+ T cells may be an effective prognostic marker for patients with CRC receiving coadministration of Endostar and PD-L1 inhibitors. Endostatin significantly improved CD8+ T-cell activity and synergistically improved the antitumor treatment effect of PD-L1 inhibitors.

## Data availability statement

The original contributions presented in the study are included in the article/supplementary material. Further inquiries can be directed to the corresponding authors.

## Ethics statement

This study was reviewed and approved by The Ethics Committee for Animal Experiment of Jinan University. Written informed consent was obtained from the individual(s) for the publication of any potentially identifiable images or data included in this article.

## Author contributions

Research idea: Y-LP and HD. Data extraction and integrated analysis: X-DC, Y-RZ, HB, SC-H, and Y-JL. Quality assessment and result interpretation: TH, HD, R-XC, and J-SH. Modification and polishing: Y-LP and HD. All authors contributed to the article and approved the submitted version.

## Funding

This research was supported by the Clinical Frontier Technology Program of the First Affiliated Hospital of Jinan University, China (No. JNU1AF-CFTP-2022-a01223), Natural Science Foundation of Guangdong Province (2019A1515011763; 2020A1515110639; 2021A1515010994; 2022A1515011695), Guangzhou Science and Technology Plan City-School Joint Funding Project (202201020084, 202201020065), and the Fundamental Research Business Expenses of Central Universities (21620306). The funding did not affect the design of the study, collection, analysis or interpretation of data or preparation of the manuscript.

## Conflict of interest

The authors declare that the research was conducted in the absence of any commercial or financial relationships that could be construed as a potential conflict of interest.

## Publisher’s note

All claims expressed in this article are solely those of the authors and do not necessarily represent those of their affiliated organizations, or those of the publisher, the editors and the reviewers. Any product that may be evaluated in this article, or claim that may be made by its manufacturer, is not guaranteed or endorsed by the publisher.
